# Incidence, therapy, and outcome in the management of chronic subdural hematoma in Switzerland: a population-based multicenter cohort study

**DOI:** 10.3389/fneur.2023.1206996

**Published:** 2023-09-14

**Authors:** Amir El Rahal, Jürgen Beck, Peter Ahlborn, Corrado Bernasconi, Serge Marbacher, Stefan Wanderer, Jan-Karl Burkhardt, Roy Thomas Daniel, Andrea Ferrari, Oliver Hausmann, Maria Kamenova, Karl Kothbauer, Katharina Lutz, Luigi Mariani, Alex Alfieri, Daniel Schöni, Philippe Schucht, Andreas Raabe, Luca Regli, Dominique Kuhlen, Martin Seule, Jehuda Soleman, Daniele Starnoni, Julien Zaldivar, Christian Zweifel, Karl Schaller, Christian Fung

**Affiliations:** ^1^Department of Neurosurgery, University Hospital of Geneva, Faculty of Medicine, Geneva, Switzerland; ^2^Department of Neurosurgery, Medical Center, University of Freiburg, Freiburg, Germany; ^3^Department of Neurosurgery, Kantonsspital St. Gallen, St. Gallen, Switzerland; ^4^Department of Neurosurgery, Bern University Hospital and University of Bern, Bern, Switzerland; ^5^Department of Neurosurgery, Cantonal Hospital Aarau, Aarau, Switzerland; ^6^Department of Neurosurgery, University Hospital Zurich, Zurich, Switzerland; ^7^Department of Neurosurgery, University Hospital Center of Lausanne, Lausanne, Switzerland; ^8^Department of Neurosurgery, Hirslanden Klinik St. Anna, Luzern, Switzerland; ^9^Department of Neurosurgery, University Hospital Basel, Faculty of Medicine, Basel, Switzerland; ^10^Department of Neurosurgery, Cantonal Hospital of Lucerne, Lucerne, Switzerland; ^11^Department of Neurosurgery, Kantonsspital Winterthur, Winterthur, Switzerland; ^12^Department of Neurosurgery, Regional Hospital Lugano (EOC), Lugano, Switzerland; ^13^Department of Neurosurgery, Cantonal Hospital Graubünden, Chur, Switzerland

**Keywords:** neurosurgery, cSDH, chronic subdural hematoma, incidence, therapy, outcome, multicentric study, management

## Abstract

**Background:**

Chronic subdural hematoma (cSDH) is a disease affecting mainly elderly individuals. The reported incidence ranges from 2.0/100,000 to 58 per 100,000 person-years when only considering patients who are over 70 years old, with an overall incidence of 8.2–14.0 per 100,000 persons. Due to an estimated doubling of the population above 65 years old between 2000 and 2030, cSDH will become an even more significant concern. To gain an overview of cSDH hospital admission rates, treatment, and outcome, we performed this multicenter national cohort study of patients requiring surgical treatment of cSDH.

**Methods:**

A multicenter cohort study included patients treated in 2013 in a Swiss center accredited for residency. Demographics, medical history, symptoms, and medication were recorded. Imaging at admission was evaluated, and therapy was divided into burr hole craniostomy (BHC), twist drill craniostomy (TDC), and craniotomy. Patients' outcomes were dichotomized into good (mRS, 0–3) and poor (mRS, 4–6) outcomes. A two-sided *t*-test for unpaired variables was performed, while a chi-square test was performed for categorical variables, and a *p*-value of <0.05 was considered to be statistically significant.

**Results:**

A total of 663 patients were included. The median age was 76 years, and the overall incidence rate was 8.2/100,000. With age, the incidence rate increased to 64.2/100,000 in patients aged 80–89 years. The most prevalent symptoms were gait disturbance in 362 (58.6%) of patients, headache in 286 (46.4%), and focal neurological deficits in 252 (40.7%). CSDH distribution was unilateral in 478 (72.1%) patients, while 185 presented a bilateral hematoma with no difference in the outcome. BHC was the most performed procedure for 758 (97.3%) evacuations. CSDH recurrence was noted in 104 patients (20.1%). A good outcome was seen in almost 81% of patients. Factors associated with poor outcomes were age, GCS and mRS on admission, and the occurrence of multiple deficits present at the diagnosis of the cSDH.

**Conclusion:**

As the first multicenter national cohort-based study analyzing the disease burden of cSDH, our study reveals that the hospital admission rate of cSDH was 8.2/100,000, while with age, it rose to 64.2/100,000. A good outcome was seen in 81% of patients, who maintained the same quality of life as before the surgery. However, the mortality rate was 4%.

## Introduction

Chronic subdural hematoma (cSDH) is a disease affecting mainly elderly individuals. The reported incidence is highly variable depending on the population and the period being studied. It ranges from 2.0/100,000 person-years in the Swedish population in 1996 to 58 per 100,000 persons-year when considering only patients who are >70 years old, with an overall incidence of 8.2–14.0 per 100,000 persons-year (1–3). Due to an estimated doubling of the population above 65 years between 2000 and 2030, cSDH will become an even more significant concern (3–5). In addition, the increasing use of oral anticoagulation has raised the incidence of cSDH (6). The growing number of patients being affected significantly impinges healthcare costs of cSDH, with current costs of ~US$ 10,000 per treatment (7, 8). Despite these facts, no evidence-based treatment strategy for cSDH has been established (9). To gain an overview of cSDH hospital admission rates, treatment, and outcome in Switzerland and to characterize the Swiss patient collective for future studies, we performed this retrospective multicenter national cohort study of patients requiring surgical treatment of cSDH.

## Materials and methods

We performed a multicenter retrospective cohort study, including patients undergoing surgical drainage of cSDH between 1 January 2013 and 31 December 2013. The neurosurgical centers included in this analysis were Swiss centers accredited for a residency program of at least 1 year of training. Almost all patients with cSDH are referred to one of those centers due to the availability of emergency services. Demographic parameters (sex and age), past medical history, presenting symptoms, and the use of anticoagulant and antiplatelet medication were recorded. Imaging at admission was evaluated according to the localization of the cSDH, and the midline shift in mm on the level of the foramen of Monro was graded according to Nakaguchi et al. into homogeneous, laminar, separated, or trabecular appearance of the cSDH (10). The therapy was divided into single- or two-burr hole craniostomy (BHC), twist drill craniostomy (TDC), and craniotomy. The outcome was assessed using the modified Rankin scale (mRS) during the outpatient clinical visit, close to 6 months after the treatment of cSDH. We defined recurrence rate as the need for reoperation and assessed the timing of recurrence in weeks after primary surgery and complications. We also evaluated clinical parameters that might contribute to poor outcomes and recurrence. Data were entered into a REDCap (2015, Vanderbilt University) database. Regional ethics committees of all the centers approved the study. Informed consent for the scientific exploration of clinical and biological data was consistent with the local ethical standards, and the Declaration of Helsinki was available to all patients. The Swiss Ethics Committees approved the study on research involving humans under the protocol “Swissethics Ec-No: 15-163.”

### Statistical analysis

Statistical analysis was performed using SPSS (IBM SPSS Statistics 25). Incidence rates are presented directly standardized using a weighted average of the stratum-specific rates. The weights were obtained using Swiss Population Distribution (Swiss age standardization). Patients' outcomes were dichotomized into good (mRS 0–3) and poor (mRS 4–6) outcomes. A two-sided *t*-test for unpaired variables was performed, while a chi-square test was performed for categorical variables. A *p*-value of <0.05 was considered to be statistically significant.

## Results

A total of 15 neurosurgical centers are accredited for neurosurgical residency programs in Switzerland, of which one was excluded because of its pure spine surgical focus. Therefore, we included patients that had been admitted to the neurosurgical department of the following 14 centers: Cantonal Hospital Aarau, University Hospital of Basel, University Hospital of Bern, University Hospital of Geneva, Cantonal Hospital Graubünden, University Hospital of Lausanne, Cantonal Hospital Lucerne, Hospital St. Anna Lucerne, Cantonal Hospital Lugano, Cantonal Hospital Sion, Cantonal Hospital St. Gallen, Cantonal Hospital Winterthur, University Hospital of Zürich, and Hirslanden clinic, Zürich.

### Patient demographics

We included 663 patients in the study with a median age of 76 years (IQR 67–83 years). Of these, 228 (34.4%) were female patients with a median age of 77 years (IQR 69–83 years), and 435 (65.6%) were male patients with a median age of 75 years (IQR 67–83 years). The female-to-male ratio was 1:1.9.

### Admission parameters and past medical history

The parameters of clinical presentation and past medical history are displayed in Table 1. Gait disturbance was the most common clinical presentation in 362 (58.6%) patients, followed by headache in 286 (46.4%) patients, focal neurological deficits in 252 (40.7%) patients, and cognitive deterioration in 246 (39.8%) patients. Multiple symptoms at presentations were seen in 505 (81.6%) patients, while 103 (16.6%) patients had one symptom, and 11 (1.8%) were asymptomatic. The median number of symptoms per patient was three (IQR 2–4). Overall median admission GCS was 15 (IQR 14–15). On admission, the median GSC of patients with unilateral or bilateral cSDH showed no significant difference (15, IQR 14–15). The distribution of anticoagulant and antiplatelet medication use is shown in Table 1. Vitamin-K antagonists were the most often used anticoagulants, prescribed in 135 (21.8%) patients. New oral anticoagulants were prescribed in nine (1.5%) patients. Aspirin was used in 169 (27.3%) patients and clopidogrel in 26 (4.2%) patients regularly before admission. Concerning past medical history, the most frequent medical condition was arterial hypertension in 367 (59.3%) patients, followed by cardiac arrhythmias in 160 (25.8%), and ischemic heart disease in 148 (23.9%) patients, respectively.

**Table 1:** Admission parameters and past medical history.

	***N* (out of)**	**%**
**Clinical presentation**		
Gait disturbance/fall	362 (618)	58.6
Headache	286 (616)	46.4
Focal neurological deficit	252 (619)	40.7
Cognitive deterioration	246 (618)	39.8
Non-specific deterioration	170 (617)	27.6
Speech disturbance	155 (619)	25.0
Acute confusion	115 (618)	18.6
Drowsiness/coma	109 (618)	17.6
Vomiting/nausea	60 (618)	9.7
Seizure	44 (617)	7.1
Incontinence	30 (615)	4.9
**Past medical history**		
Hypertension	367 (619)	59.3
Arrhythmia	160 (619)	25.8
Ischemic heart disease	148 (619)	23.9
Diabetes mellitus	96 (619)	15.5
Renal insufficiency	78 (619)	12.6
Dementia	78 (619)	12.6
Cerebrovascular accident	75 (619)	12.1
DVT/PE	54 (619)	8.7
COPD	29 (619)	4.7
Hepatic insufficiency	22 (619)	3.6
**Medication**		
Aspirin	169 (618)	27.3
Clopidogrel	26 (618)	4.2
Vitramin-K antagonist	135 (619)	21.8
New OAK	9 (618)	1.5

Gait disturbance is the most prevalent symptom, and hypertension is the most common comorbidity. Anticoagulation and antiplatelet drugs were administered, respectively, by 31.5 and 22.3% of the patients.

COPD, chronic obstructive pulmonary disease; DVT, deep vein thrombosis; PE, pulmonary embolism.

### Radiological characteristics

In total, 478 (72.1%) patients presented with a unilateral cSDH, of which 243 (50.1%) affected the left side and 235 (49.2%) the right side, whereas in 185 patients, a bilateral hematoma was present. A median midline shift of unilateral cSDH was 7.0 mm (IQR 4–11 mm), compared to 3 mm (IQR 0–5 to 6 mm) of bilateral cSDH (*p* < 0.001). The hematoma was homogeneous in 308 patients (59.8%), laminar in 75 patients (14.6%), separated in 132 patients (25.6%), and trabecular in 171 (33.2%) cases.

### Incidence of cSDH

**Figure 1:**
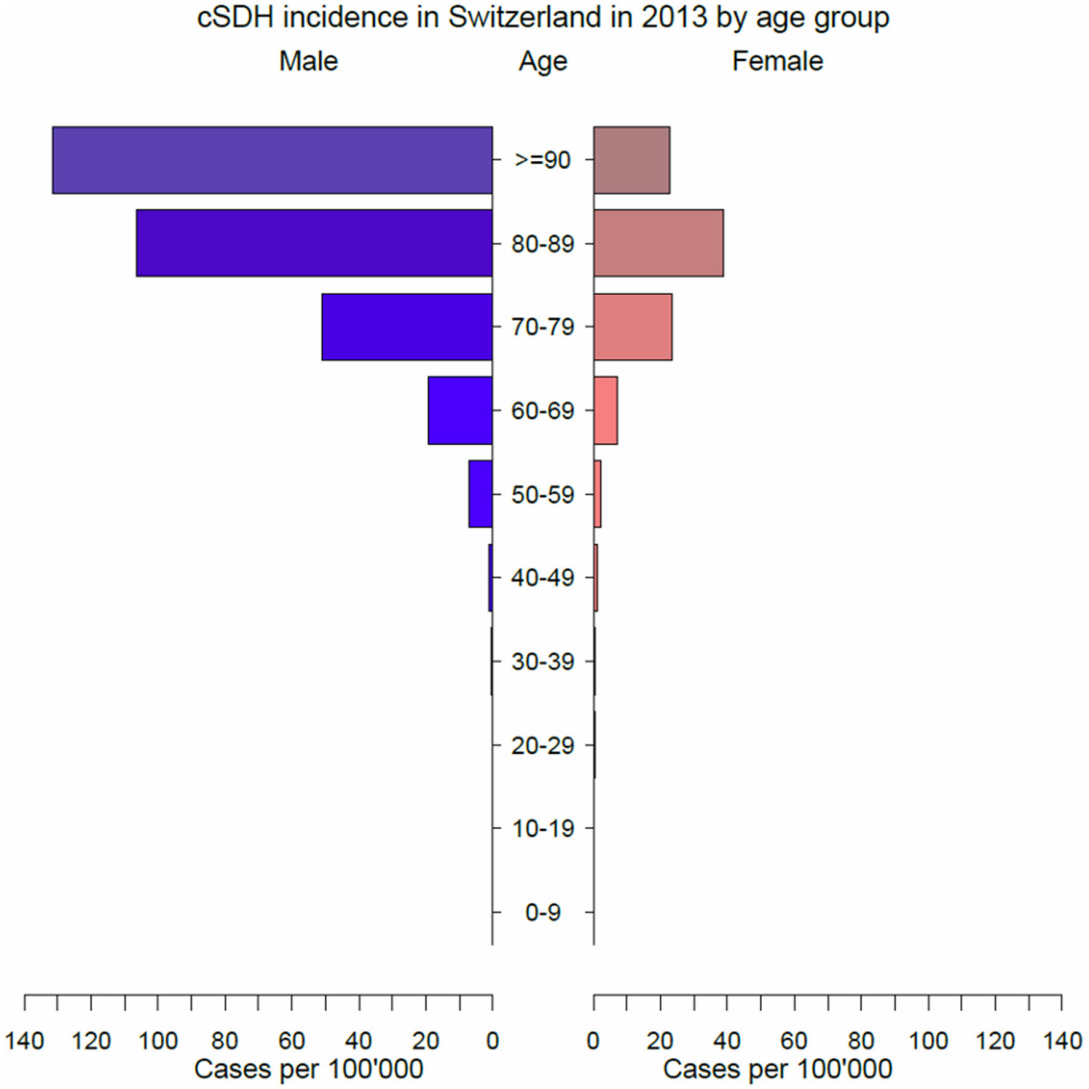
Incidence rate of cSDH per age group and sex. The highest incidence rate for women is in the age group 80–89 years, while for men, it is in the 70–79 years age group.

The overall incidence rate was 8.2/100,000 persons, 10.8 for men and 5.5 for women. Absolute numbers and incidences of cSDH per age group and sex are displayed in Table 2 and Figure 1. The highest absolute number of cSDH in men occurred in the age group of 70–79 years, whereas in women, it was in the age group of 80–89 years. The incidence of cSDH rose steadily to 131.5/100,000 in men who were 90 years or older. In women, the highest incidence rate was in the age group of 80–89 years. Overall, the highest incidence rate was 64.2/100,000 persons in patients 80–89 years old.

**Table 2:** Absolute numbers of cSDH per age group and sex.

	**Absolute numbers**			**Incidences**		
**Age group**	**Male**	**Female**	**Total**	**Male**	**Female**	**Total**
0–9	0	0	0	0	0	0
10–19	1	1	2	0.2	0.2	0.2
20–29	0	2	2	0	0.4	0.2
30–39	3	2	5	0.5	0.4	0.4
40–49	7	8	15	1.1	1.3	1.2
50–59	42	13	55	7.2	2.3	4.8
60–69	84	33	117	19.4	7.3	13.2
70–79	141	78	219	51.1	23.6	36.1
80–89	133	80	213	106.4	38.7	64.2
≥90	24	11	35	131.5	22.6	52.4
Total	435	228	663	10.8	5.5	8.2

The overall incidence rate is 8.2/100,000 people in Switzerland. However, in men, the incidence of cSDH rises steadily to 131.5/100,000 in men who are 90 years or older.

### Treatments

The most often used surgical technique as primary treatment for cSDH was BHC. BHC was performed for 758 (97.3%) cSDH cases, of which 166 (21.9%) were 1-BHC, and 592 (87.1%) were 2-BHC. There were 20 patients (2.6%) who were treated by craniotomy, and one was treated using TDC. Additionally, data on drains was available for 600 patients, and 756 patients were operated for cSDH. Subdural drains were inserted during 261 (33.2%) evacuations, and subgaleal drains were inserted during 452 (57.2%) evacuations. In 43 evacuations, no drains were inserted postoperatively. Data for recurrent hematoma was available for 518 (78.1%) patients, of which 104 patients (20.1%) experienced a recurrence after a median of 2 weeks (IQR 1–4 weeks). There were 17 patients (2.6%) who experienced a second recurrence after a median of 3 weeks (IQR 1–10) after surgical drainage of the first recurrence.

### Outcome

**Figure 2:**
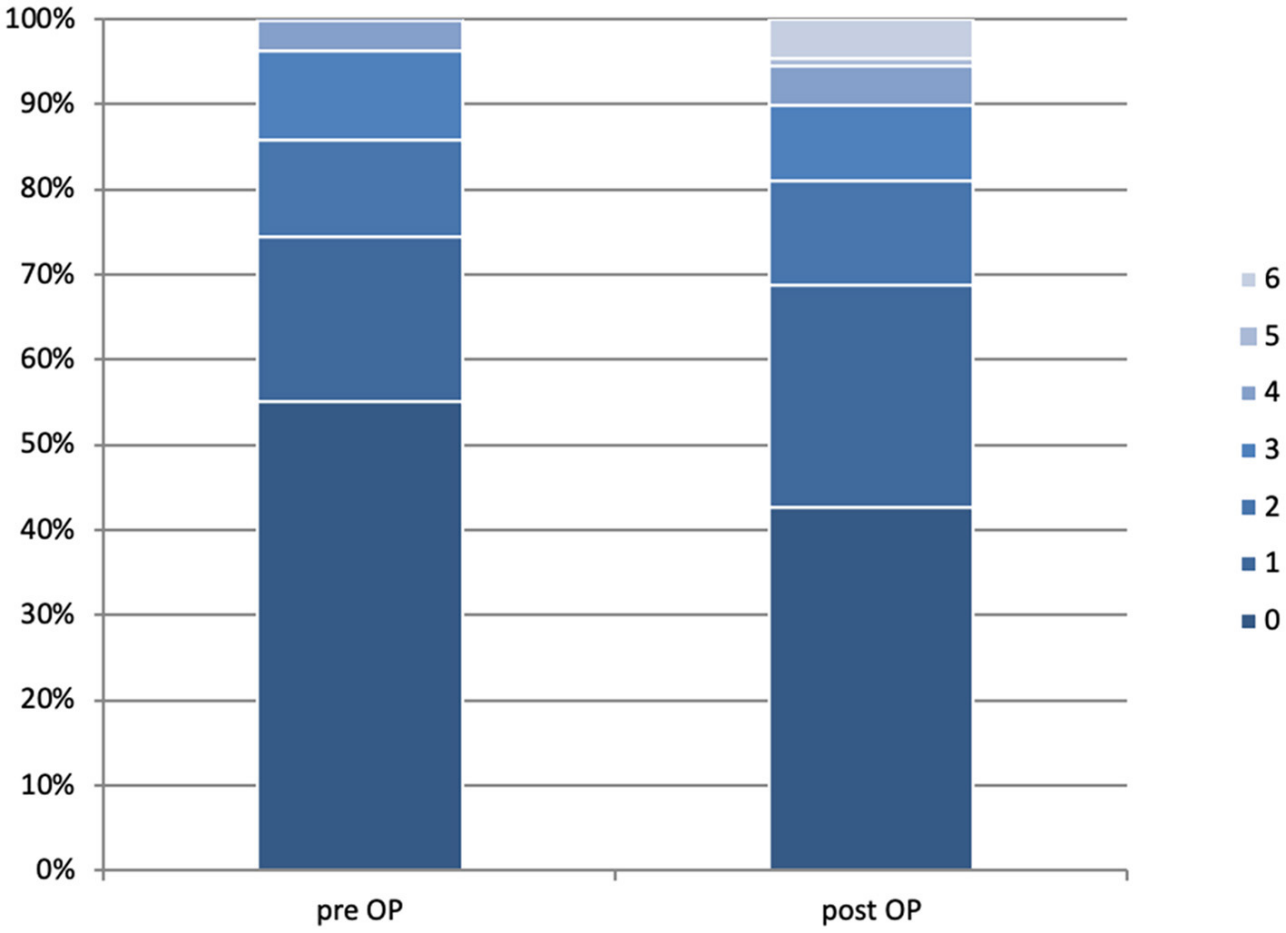
Clinical status of patients according to the modified Rankin scale before **(left)** and after **(right)** surgical treatment of their chronic subdural hematoma. The bars represent all assessed patients at respective time points and are set to 100%. We evaluated 615 patients, and 580 were pre-operatively available for the postoperative follow-up assessment. The median time of follow-up assessment was 2 months.

Before the onset of symptoms related to the cSDH, approximately half of the patients presented with an mRS of 0 (*n* = 339, 55.1%). In total, 119 patients had an mRS of 1 (19.3%), 70 patients had an mRS of 2 (11.4%), 64 patients had an mRS of 3 (10.4%), 22 patients had an mRS of 4 (3.6%), and one patient had an mRS of 5 (0.2%). After treatment of cSDH, 247 patients presented with an mRS of 0 (42.6%), 151 patients had an mRS of 1 (26%), 70 patients had an mRS of 2 (12.1%), 54 patients had an mRS of 3 (9.3%), 26 patients had an mRS of 4 (4.5%), five patients had an mRS of 5 (0.9%), and 27 patients (4.7%) presented an mRS of 6 (*p* < 0.001) (Figure 2). Median time of outcome assessment was 2 months (IQR 1–6 months) postoperatively. Factors associated with poor outcomes were age, GCS on admission, mRS on admission, and the occurrence of multiple deficits present at the diagnosis of the cSDH. Factors not showing significant association with poor outcome were midline shift, neither in uni- nor in bilateral cSDH, sex, the use of antiplatelet medication or oral anticoagulation, uni- or bilateral cSDH, or the side of cSDH.

## Discussion

To the best of our knowledge, this is the first study to evaluate hospital admission rates of cSDH based on a multicenter national cohort including all age groups and all reference treating hospitals. We have shown that the treatment of patients with chronic subdural hematoma in Switzerland was relatively uniform, with a high rate of burr hole evacuation and a high rate of postoperative drainage placement.

### Incidence

Our study yielded a hospital admission rate of cSDH of 8.2/100,000 person-years. This aligns with previous studies reporting an incidence of 8.2–14 per 10,000 person-years (1–3, 11). Previous studies have assessed the incidence in confined areas or defined patient collectives (1, 2, 12, 13). The definition of the incidence of cSDH is challenging since many more patients probably present a mild course with spontaneous resolution without presentation to medical care (12). Others might present subdural hygroma with or without transformation into a classical cSDH or are hospitalized without surgical care. Reports about the rate of spontaneous resolution or conservatively treated cSDH are scarce primarily since clinically silent cSDH is not in the physician's attention and symptomatic cSDH is generally treated surgically (14–17). Medically treated patients are limited to small case series. In a recent study by Baiser et al., only 29% of newly diagnosed cSDH required surgical drainage (17–20). Transferred to our results, this would mean a 3-fold increase in the incidence of cSDH in Switzerland. Nevertheless, in the recent trial of dexamethasone for chronic subdural hematoma in symptomatic patients, 94% underwent surgery to evacuate their hematomas (21). This illustrates the difficulties in assessing the incidence of cSDH. A new finding of this study is the reported peak incidence of 131.5 per 100,000 person-years in men ≥ 90 years. Our study shows a steady incidence increase with increasing age up to 89 years. Higher incidences with increasing age have been reported earlier, without reaching an incidence rate of >130/100,000 person-years.

### Treatment

The preferred primary treatment of choice was BHC (*n* = 758). Only a minority of patients were treated by craniotomy (*n* = 20) or TDC (*n* = 1). The type of surgical treatment was at the discretion of the treating surgeon. The amount of performed BHC aligns with the results of a previous systematic review by Weigel et al., which states that BHC has a comparable low morbidity rate as TDC but a much lower recurrence rate (22). BHC is also the most common form of treatment in other countries. In 2009, Santarius et al. concluded that the drain's subdural insertion reduces the recurrences rate from 24 to 9.3% and the mortality from 18.1 to 8.6% (23). In our series, we inserted a total of 713 drains (261 subdural and 452 subgaleal). The rate of drain placement in Switzerland (>90%), either subdural or subgaleal, is interestingly high compared to the 80% rate found in the international survey by Soleman et al., and this might be attributable to the randomized controlled trial done by Santarius et al. in 2009, which showed a significant benefit in reducing recurrence rates following the use of drains after a burr hole evacuation of cSDH (23). Moreover, Soleman et al. (24) concluded in a recent RCT that subperiosteal drain insertion led to lower recurrence rates, fewer surgical infections, and lower drain misplacement rates compared to subdural drains (25). Häni et al. concluded in a prospective trial that the placement of subgaleal drains rather than subdural drains did not increase the risk of cSDH recurrence. The outcome was also assessed and comparable in both groups (26).

### Outcome

**Figure 3:**
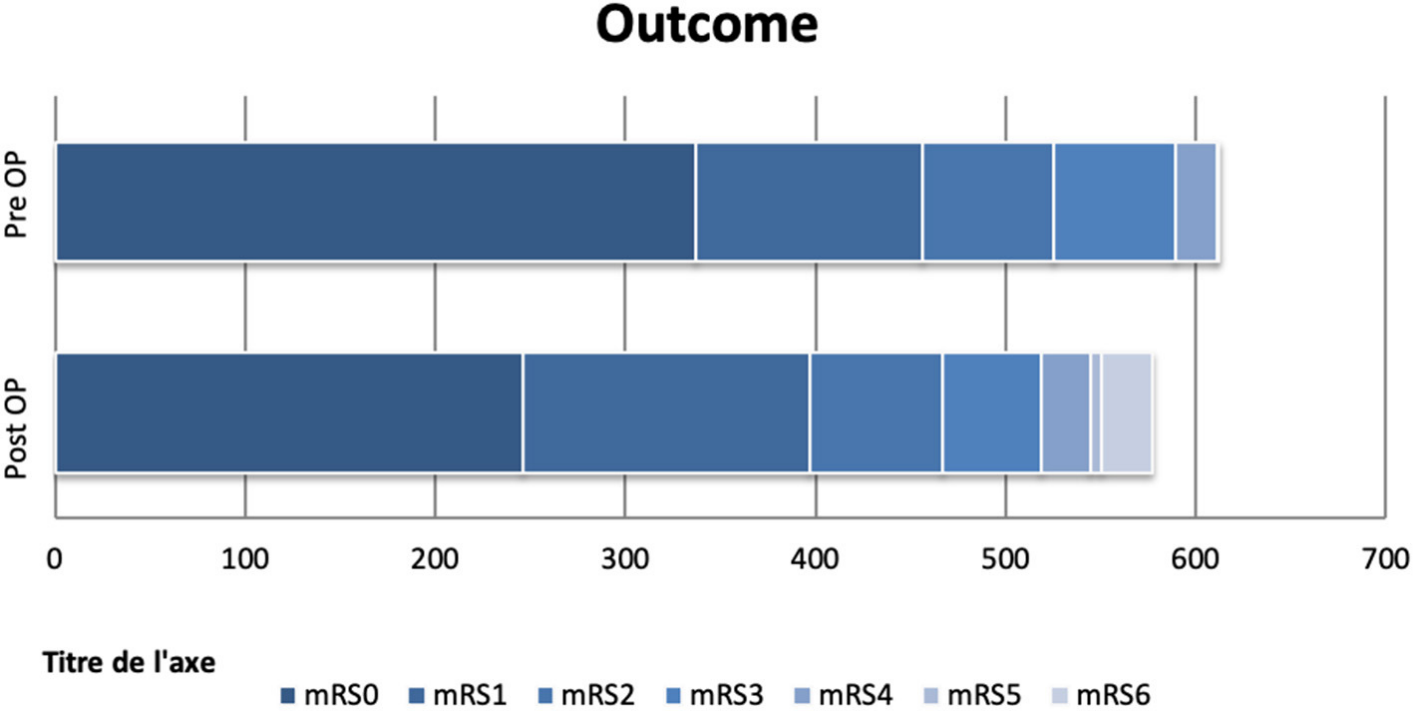
Clinical status of patients according to the modified Rankin scale in absolute numbers. The top row represents 615 preoperative assessed patients, and the bottom row 580. Good outcomes (mRS <3) are seen in almost 81% of patients treated surgically. The outcome was influenced by age, GSC on admission, mRS on admission, and occurrence of multiple neurological deficits at the sickness onset.

We dichotomized the outcome of our patients into favorable outcomes, with an mRS of 0–3, and poor outcomes, with an mRS of 4–6. Higher age, lower GCS and higher mRS on admission, and occurrence of multiple deficits were associated with poor outcomes (Figure 3). This confirms the data observed by Santarius et al. in 2009, where mRS on admission and neurological deficits were strong predictors of a bad outcome. GCS on admission indirectly reflects the patient's neurological status, confirming the significance of these factors and may favor an early surgery before the appearance of neurological deterioration.

No difference was found between the side of the hematomas (right/left) and the outcome of patients, with 31 patients having a bad outcome with left-sided hematomas and 37 with right-sided hematomas. No difference was found between subdural vs. subgaleal drains. Santarius et al. conducted their randomized controlled study based on the insertion of subdural drains with the conclusion of a lower recurrence rate. Zhang et al. showed no difference between subdural and subgaleal drains in a retrospective study of 570 patients, which was confirmed by the recent Swiss study conducted by Häni et al. (26, 27). In our cohort, 104 patients (20.1%) experienced a recurrence after a median of 2 weeks (IQR 1–4 weeks) and 17 patients (2.6%) experienced a second recurrence after a median of 3 weeks (IQR 1–10) after surgical drainage of the first recurrence. The usefulness of routine follow-up CT to predict symptomatic recurrence is also questionable and has been recently studied. Schucht et al. showed no benefit for routine follow-up CT after surgery for cSDH, and repeated surgery was fewer in the clinically followed patients (28).

Modern treatment strategies have recently been explored to manage chronic subdural hematoma (CSDH), including middle meningeal artery (MMA) embolization (29, 30). MMA embolization has shown promising results as an option for recurrent or refractory CSDH (31). Link et al. showed in 2018 that MMA embolization may reduce the recurrence rate and the need for repeated surgeries (32). More extensive research is needed to validate these findings and assess the long-term outcomes.

An additional facet of CSDH management has involved the application of corticosteroids, notably dexamethasone (20). Conclusions drawn from systematic reviews suggest that glucocorticoids may be safe and effective in diminishing the chances of recurrence when used supplementary to surgical intervention. Furthermore, they could serve as independent treatment options to avoid the need for surgical procedures (33–35). Nonetheless, the findings from a randomized, multicenter, placebo-controlled study, which sought to evaluate the impact of dexamethasone on patients suffering from symptomatic chronic subdural hematoma, painted a different picture. The results demonstrated that dexamethasone led to fewer positive outcomes and a higher frequency of undesirable events than placebo at the 6-month mark. As a result, this treatment has since been curtailed in routine medical practice weighing the potential benefits against the risks (21).

This new insight into the effectiveness of dexamethasone underscores the importance of continuous research to evaluate and refine our treatment strategies for cSDH.

### Mortality

In our study, the mortality rate at 15 weeks was 4%, equating to 27 patients. The rate was lower than the rate reported by Lukasiewicz et al. in 2016, on 759 patients, which was 17% at 30 days, but consistent with the 4% mortality rate found by Almenawer et al. in a systematic review and meta-analysis of 34,829 patients in 2014 (11). Compared to the recent randomized controlled studies performed in the UK, our cohort's mortality rate was lower. The reported rate by Santarius et al. in 2009 was 8.6% in the drainage group and 18.1% in the non-drainage group, and in the recent study performed by Hutchinson et al., the mortality rate reported was 8.8% in the dexamethasone group and 5% in the placebo group at 6 months. Schucht et al. reported a mortality rate of 5.4% of patients in a recent randomized control trial (28). Lukasiewicz et al. considered only the patients treated by craniotomy and probably included acute subdural hematomas explaining the high mortality (36). In our study, the preferred primary treatment was BHC (*n* = 758). Only a minority of patients were treated by craniotomy (*n* = 20).

## Limitations of the study

Our study is limited by the retrospective design. Conservatively treated patients were omitted, and only some might have been treated in private hospitals, minimally impacting our data. Moreover, the study was conducted in a high-income country, which is a factor to consider.

## Conclusion

As the first multicenter national cohort-based study analyzing the disease burden of cSDH, our study reveals that the hospital admission rate of cSDH was 8.2/100,000. It increased with age up to 64.2/100,000 persons in patients aged 80–89 years. The mortality rate was 4%, which is lower than the literature reports. Good outcome was seen in almost 81% of the patients but was negatively influenced by higher age, lower GCS on admission, higher mRS on admission, and occurrence of multiple neurological deficits present at the diagnosis of the cSDH.

## Data availability statement

The datasets generated and analyzed during this study are not publicly available since their content may compromise the privacy of the research participants. Datasets are available from the corresponding author upon reasonable request.

## Ethics statement

The studies involving human participants were reviewed and approved by Swissethics (Ec-No: 15-163). The patients/participants provided their written informed consent to participate in this study.

## Author contributions

CF, AE, AR, and KS: study design. AE, PA, SM, SW, J-KB, AF, OH, MK, KL, DSc, MS, JS, DSt, JZ, CZ, and CF: data acquisition. AR, LM, LR, KS, DK, and RD: infrastructure. CB, AE, and CF: data analysis. AE and CF along with contributions from all authors: manuscript writing. All authors contributed to manuscript corrections. All authors contributed to the article and approved the submitted version.

## In memoriam

Prof. Karl Kothbauer, who contributed to the realization of the manuscript as the past Clinical Director of the Cantonal Hospital of Lucern.
